# Supplementary material for: No Object–Location Memory Improvement through Focal Transcranial Direct Current Stimulation over the Right Temporoparietal Cortex
Source: Life (Basel). 2024 Apr 23;14(5):539. doi: 10.3390/life14050539 (PMC11122124; doi:10.3390/life14050539)
Supplement: Supplementary file 1 [file life-14-00539-s001.zip › life-2879916-supplementary.pdf]

Supplementary Table S1. Descriptive values from the object-location memory training

		Group	Number of cases	Mean [%]	95%-CI	
Day 1	Recall 1	anodal	26	11.1	7.5	14.6
		sham	26	15.2	11.4	19.0
	Recall 2	anodal	26	27.9	22.1	33.7
		sham	26	35.3	28.1	42.4
	Recall 3	anodal	26	44.8	37.0	52.7
		sham	26	54.0	44.5	63.6
Day 2	Recall 4	anodal	26	40.8	33.4	48.1
		sham	26	50.2	40.4	60.0
	Recall 5	anodal	26	55.9	47.2	64.6
		sham	26	66.9	57.2	76.6
	Recall 6	anodal	26	67.6	59.5	75.7
		sham	26	76.8	66.8	86.9

Note. CI. Confidence Interval. Mean displays number of correct responses during each recall trial in percent.

Supplementary Table S2. Linear mixed model

	Estimate	2.5 % CI	97.5 % CI	p-value
(Intercept)	0.05	-0.44	0.54	0.85
Group (anodal)	-0.04	-0.14	0.06	0.44
Recall	0.19	0.17	0.22	< 0.001
Day	0.36	0.32	0.40	< 0.001
Baseline	0.29	0.02	0.55	0.05
Age	0.00	-0.02	0.02	0.97
Sex (female)	0.04	-0.07	0.15	0.50
Strategy (Position)	-0.10	-0.21	0.00	0.07
Group (anodal):Recall	-0.03	-0.06	0.01	0.14
Group (anodal):Day	-0.06	-0.12	0.01	0.08
Recall:day	-0.06	-0.09	-0.03	0.00
Group (anodal):Recall:Day	0.03	-0.02	0.07	0.28

Note. CI. Confidence interval. Linear mixed model for analyzing primary outcome, random intercept for participant. Performance ~ Group * Recall * Day + Baseline + Age + Sex + Strategy + (1 | ID). Number of observations: 312, groups: ID, 52
